# Which Symptoms of Nomophobia, Social Networking Site Addiction, and Fear of Missing Out (FoMO) Directly Affect Mental Health? A Symptom Network and Flow Analysis Study

**DOI:** 10.1002/pchj.70068

**Published:** 2025-11-28

**Authors:** Xiaofan Zhang, Jiashuo Zhang, Feihu Yao, Peipei Cao, Sipu Guo, Shengzhi Liu

**Affiliations:** ^1^ Department of Information Management Peking University Beijing China; ^2^ Peking University Publishing Research Institute Peking University Beijing China; ^3^ Subsidiary High School of Taiyuan Normal University Taiyuan China; ^4^ Library of Beijing Administration Institute Beijing China; ^5^ Institute of Developmental Psychology, Faculty of Psychology Beijing Normal University Beijing China; ^6^ School of Digital Media and Design Arts, Faculty of Communication Beijing University of Posts and Telecommunications Beijing China

**Keywords:** anxiety, depression, fear of missing out, network analysis, nomophobia, social networking site addiction

## Abstract

Nomophobia, social networking site (SNS) addiction, and fear of missing out (FoMO) are increasingly recognized as interrelated digital‐age phenomena that pose risks to young people's mental health. However, limited research has examined how specific symptoms across these domains interact and contribute to anxiety and depression. This study aims to make a novel contribution by applying network and flow analysis to uncover the symptom‐level interconnections among nomophobia, SNS addiction, FoMO, and their links to mental health outcomes. A total of 3108 college students completed validated scales measuring SNS addiction, FoMO, nomophobia, anxiety, and depression. Gaussian graphical models and centrality indices were used to estimate symptom networks. Flow networks were constructed to identify pathways connecting symptoms to mental health outcomes. Strong intranetwork associations were found within all three domains. “FoMO on information” emerged as the most central and influential bridge symptom, connecting nomophobia and SNS addiction. Flow network analysis revealed that “FoMO on information” was also the strongest individual predictor of both anxiety and depression. Other symptoms, such as “fear of losing internet connection” and “SNS‐related insomnia,” also showed notable associations with mental health outcomes. These findings highlight the potential of network and flow analysis to identify transdiagnostic mechanisms across digital behavioral addictions. “FoMO on information” appears to be a key symptom linking nomophobia and SNS addiction and may represent a promising target for interventions aimed at reducing comorbid anxiety and depression among adolescents.

## 1 Introduction

Nomophobia, a portmanteau of “no mobile phone phobia,” refers to the fear or anxiety experienced when individuals are unable to maintain contact via their mobile devices (Mail Online 2008), which stems from the fact that smart devices have become a routine for a growing number of individuals to obtain information, communicate, and fulfill their daily work through social media (Thompson 2024). Individuals with nomophobia may experience the inability to communicate, perceived disconnection from social networks, limited access to information, and fear of missing important information afforded by mobile phone use (Yildirim and Correia 2015). A longitudinal study analyzed data from 4285 children (mean age = 10 years) and identified that nearly one‐third of participants exhibited an increasing trajectory of addictive use of social media or mobile phones beginning at age 11, highlighting a significant upward trend in screen‐related behavioral dependency among adolescents (Xiao et al. 2025). In one study focusing on adolescent students, the majority (76.7%) were found to exhibit high levels of nomophobia (Maghaireh et al. 2025). Recently, a meta‐analysis of 43 studies relevant to nomophobia in 18 countries shows that the overall prevalence of nomophobia is about 94%, and the prevalence for adolescents is about 90% (Al‐Mamun et al. 2025).

Furthermore, nomophobia has been linked to maladaptive behaviors and cognitive disruptions, including self‐blame, distraction, and denial, which may hinder attentional functioning (Bragazzi et al. 2019). A rich body of studies has indicated that social networking site (SNS) addiction and fear of missing out (FoMO) are two major risk factors for nomophobia (Gaber Hamzaa et al. 2024; Kuss and Griffiths 2017; Lin and Huang 2025). As a result, nomophobia has been shown to significantly impact adolescents' mental health, often manifesting in increased levels of anxiety (Zhu et al. 2024) and depression (Caba‐Machado et al. 2024). However, despite extensive research linking nomophobia with FoMO and SNS addiction, few studies have clearly examined their interplay at the symptom level or how these interactions may contribute to common mental health outcomes such as anxiety and depression.

### 1.1 The Bidirectional Relationship Between SNS Addiction and Nomophobia

SNS addiction refers to an excessive preoccupation with SNSs, driven by a strong urge to use them, to the extent that it disrupts daily functioning, including social life, academic or work performance, interpersonal relationships, and well‐being (Andreassen and Pallesen 2014). As behavior‐like addictions, adolescents are more prone to be addicted to smartphones (Kwon et al. 2013), problematic social media use (Tung et al. 2025), and SNS addiction (Kuss and Griffiths 2011), which may lead to mental disorders (Chen et al. 2020), such as anxiety (Ranjan et al. 2021) and depression (Lin et al. 2016).

Drawing on self‐determination theory (SDT), we propose a unified conceptual model to explain the interplay among nomophobia and SNS addiction in affecting mental health. According to the SDT, individuals with low satisfaction of these basic needs tend to use more SNS (Przybylski et al. 2013), which may satisfy the needs for belonging, popularity, and autonomy through more self‐presentation, communication, and feedback (Beyens et al. 2016; Ryan and Deci 2000). Meanwhile, as stated by the basic psychological needs theory (Vansteenkiste et al. 2020), limited offline social opportunities in schools and junior high students rely on mobile devices to meet basic relatedness needs. What is more, social media uses immediate rewards to maintain interest and encourage involvement, providing free and open spaces for adolescents to express themselves, thus increasing their willingness and autonomy to use social media (Anshari et al. 2019). Over time, this phenomenon may lead to nomophobia and problematic social networking use, characterized by fear of disconnection and missing online interactions (Milyavskaya et al. 2018). The cross‐sectional evidence indicates that nomophobia is positively correlated with separation anxiety and social phobia (Kuscu et al. 2021), and SNS addiction (Hashemi et al. 2023) in adolescents and also predicts SNS addiction (Lin et al. 2021). Increased time spent on smartphones or SNS platforms has been strongly linked to higher levels of nomophobia (Kaviani et al. 2020).

Importantly, extrinsic motivations for SNS use, such as the desire for recognition or external validation, may result in increased depression and anxiety (Kaviani et al. 2020). Empirical evidence shows that SNS addiction is associated with depression, lower self‐esteem, anxiety, and loneliness (Chao et al. 2023; Wang et al. 2025). Caba‐Machado et al. (2024) also found that nomophobia has a direct impact on adolescents' levels of anxiety, stress, and depression. Despite these findings, limited research has explored the symptom‐level dynamics between SNS addiction and nomophobia, particularly in identifying which symptoms are most strongly associated with mental health outcomes.

### 1.2 The Bidirectional Relationship Between FoMO and Nomophobia

*FoMO* refers to the constant worry that others are enjoying rewarding experiences without individuals (Milyavskaya et al. 2018; Roberts and David 2020). According to the compensatory Internet use theory (Kardefelt‐Winther 2014), individuals are closely intertwined with the use of mobile phones and other digital media in their daily lives. Such usage can facilitate daily functioning and serve as a psychological buffer against negative emotions (Przybylski et al. 2013). In some way, individuals use social media as a coping mechanism for emotional regulation, which may also have unintended long‐term consequences. Specifically, individuals may become increasingly preoccupied with the FoMO on important information and worry about not being able to stay updated with online content in real time (Tandon et al. 2021). This persistent concern can ultimately lead to nomophobia (Alhaj et al. 2024; Hoşgör and Gündüz Hoşgör 2019). Cross‐sectional evidence indicates that FoMO is associated with nomophobia, particularly in relation to interpersonal sensitivity and paranoid ideation (Yılmaz and Bekaroğlu 2022). However, this relationship appears to be more complex and context‐dependent (Wen et al. 2023). Specifically, FoMO influences users' behavior and health as they have to frequently check their smartphones to stay connected and avoid missing any important information (Elhai et al. 2016). Based on the social comparison theory, individuals always compare self and others to conduct self‐assessment and self‐enhancement (Crusius et al. 2022). SNSs provide abundant opportunities for comparison, so individuals are addicted to comparing upward and downward through the information (Vogel et al. 2014). Therefore, individuals are afraid of missing any information and thus fear losing their smartphones (Alt 2015), which is the major manifestation of nomophobia. In summary, compensatory Internet use theory and social comparison theory both offer an explanatory understanding of the relation between nomophobia, FoMO, and SNS addiction, as well as their relation to how they affect adolescents' mental health.

### 1.3 Network Analysis and the Current Study

FoMO has been identified as a core risk factor for both SNS addiction (Li et al. 2024) and nomophobia (Gaber Hamzaa et al. 2024). Their complex and interconnected nature may jointly contribute to the development of mental health problems in adolescents. However, most existing studies have not examined these relationships from a symptom‐level perspective. Network analysis conceptualizes individual symptoms as nodes and the relationships between them as edges, allowing researchers to examine how symptoms interact within a connected system (Bringmann et al. 2013), offering a novel perspective for exploring the complex relationships among nomophobia, FoMO, and SNS addiction. Using the network analysis approach, we can identify key bridge symptoms (i.e., the sum of the edge weights of a node within its community that connects to other nodes in a different mental disorders community; Jones et al. 2021) that connect these three psychological constructs.

Therefore, this study pursues two aims:

Aim 1: Examine the network‐level relationships among nomophobia, SNS addiction, and FoMO, and identify bridge symptoms that can bridge this comorbidity of the network.

Aim 2: Identify the specific symptoms within this interconnected network that are most directly associated with anxiety and depression, with the goal of informing more targeted psychological interventions.

## 2 Method

### 2.1 Participants and Procedure

The study was conducted in March 2025 across Shanxi and Fujian Provinces in China via an online survey platform (https://www.wjx.cn) through four universities using a purposive sampling approach. Initially, a total of 3865 college students whose ages ranged from 18 to 26 years were recruited. After excluding 757 students who disagreed with participating in the current research, the final data analysis included 3108 college students (males, 48.8%; age mean ± SD = 20.75 ± 1.61).

Before completing the questionnaires, all participants were informed about the purpose of the study and provided electronic informed consent. This study received ethical approval from the Ethics Committee of Peking University (Number: BUPT‐P‐2025017).

### 2.2 Measures

#### 2.2.1 Short Version of SNS Addictive Tendencies Scale

The short version of the SNS addictive tendencies scale (Dai and Kong 2022; Milošević‐Đorđević and Žeželj 2014) was used to measure the SNS addiction of individuals. The scale consists of six items that measure six common components of SNS addiction, including “declining productivity,” “insomnia,” “dual existence,” “social network addiction,” “online relationship satisfaction,” and “virtual friend anxiety.” Participants rated their agreement on a five‐point Likert scale (1 = *strongly disagree*, 5 = *strongly agree*), with higher scores indicating more severe SNS addiction. In the current study, Cronbach's *α* of this scale was 0.89.

#### 2.2.2 FoMO Scale

The FoMO scale has eight items that measure individuals' FoMO on information (four items, e.g., “I'm afraid that others are having more exciting experiences or gaining more than I am.”) and situation (four items, e.g., “I feel upset when I miss the chance to meet up with my friends.”) (Li et al. 2019). The FoMO scale was a five‐point Likert‐type scale, ranging from one (“Not at all true”) to five (“Completely true”), with higher scores indicating stronger FoMO. In this study, Cronbach's *α* for the whole FoMO scale was 0.92, while it was 0.92 for the information subscale and 0.87 for the situation subscale, respectively.

#### 2.2.3 Nomophobia Questionnaire

Nomophobia was measured using the Nomophobia Questionnaire (Ren et al. 2020). The questionnaire consisted of 16 items, with four items forming each dimension. The four dimensions were (1) fear of being unable to access information (e.g., “I would feel uncomfortable without constant access to information through my smartphone.”; Cronbach's *α* = 0.93); (2) fear of losing convenience (e.g., “Running out of battery in my smartphone would scare me.”; Cronbach's *α* = 0.93); (3) fear of losing contact (e.g., “I get worried when my family and/or friends can't reach me because my phone isn't with me.”; Cronbach's *α* = 0.95); and (4) fear of losing internet connection (e.g., “I feel anxious when being separated from my phone disconnects me from the internet.”; Cronbach's *α* = 0.97). Participants rated their agreement on a seven‐point Likert scale (1 = *strongly disagree*, 7 = *strongly agree*), with higher scores indicating stronger nomophobia. In this study, the Cronbach's *α* for the whole questionnaire was 0.98.

#### 2.2.4 Generalized Anxiety Disorder 7‐Item Scale (GAD‐7)

GAD‐7 was used to assess the anxiety symptoms (Spitzer et al. 2006). Participants were asked to evaluate how often they experienced anxiety symptoms (e.g., “anxiousness,” “restlessness,” and “irritability”) in the past 2 weeks. Responses were rated on a four‐point Likert scale (0 = *Not at all*, 3 = *almost every day*), with higher scores indicating severe anxiety. In this study, the Cronbach's *α* for GAD‐7 was 0.96.

#### 2.2.5 Patient Health Questionnaire‐9 (PHQ‐9)

PHQ‐9 was used to assess the depression symptoms (Kroenke et al. 2001). Participants evaluated their feelings of anxiety symptoms (e.g., “anhedonia,” “depressed mood,” and “guilt”) in the past 2 weeks on a four‐point Likert scale (0 = *Not at all*, 3 = *almost every day*). Higher scores indicated severe depression. In this study, the Cronbach's *α* for PHQ‐9 was 0.95.

#### 2.2.6 Parental Marital Status and Marriage Quality

Parental marital status (Niu et al. 2023) was assessed using a single item (i.e., “What is the current marital status of your parents?”; 1 = *Married and living together*, 5 = *Divorced, at least one parent has remarried*). According to the previous study (Tao, Niu, et al. 2024), the quality of parental marriage was also assessed using a single item (i.e., “How well do your parents communicate with each other?”; 1 = *Harmonious relationship, never argue*, 5 = *Constantly argue, almost every day*).

### 2.3 Statistical Analysis

Data were analyzed using R software (version 4.3.2; R Core Team 2023). First, descriptive analyses were performed to summarize participants' demographic characteristics and key study variables (e.g., SNS addictive tendencies, FoMO, nomophobia, anxiety, and depression). Independent sample *t* tests were conducted to explore differences in these variables between male and female students, as well as between only‐child and nononly‐child participants. Moreover, correlational analyses were performed to explore associations between key study variables and demographic information, such as age, parental marital status, and parental relationship quality. Then, we conducted the following network analysis.

#### 2.3.1 Network Structure and Centrality Estimation

The network of FoMO, SNS addictive tendencies, and nomophobia was estimated and visualized using the R packages *bootnet* 1.4.3 and *qgraph* 1.6.9 (Epskamp et al. 2018, 2012). The network was modeled using the estimateNetwork function using a Gaussian Graphical Model (GGM) graphical least absolute shrinkage and selection operator (LASSO) method. Nodes represent the dimensions or items of the variables, and edges represent partial correlations between them. Blue solid edges indicate positive correlations and red dashed edges indicate negative correlations; thicker edges denote stronger associations (Borsboom and Cramer 2013).

The Expected Influence (i.e., EI; the sum of all edges extending from a given symptom) and bridge EI centrality were computed for each node using the *centralityPlot* function (Opsahl et al. 2010). EI reflects the symptom's overall influence on the network. Bridge EI highlights its bridging role (Jones et al. 2021). Following prior studies, we focused on nodes with standardized centrality values exceeding 1 (Tang et al. 2023).

Additionally, we estimated an additional network that included demographic covariates to rule out potential confounding effects of demographic information. We extracted the submatrix of primary analytic variables from this covariate‐adjusted network and used Wilcoxon tests to compare it with the corresponding matrix from the original network (Tao, Zou, et al. 2024).

#### 2.3.2 Network Stability and Accuracy

The accuracy and stability of the network were assessed using the R package *bootnet* 1.4.3 (Epskamp et al. 2018). A bootstrapping test for the edges was first performed to compute 95% confidence intervals (CIs) for edges, with greater overlap and narrower CIs indicating higher accuracy. Then, we conducted a case‐dropping bootstrap procedure to estimate the correlation stability coefficient (CS‐C), an index reflecting the stability of centrality estimates. The CS‐C measures the correlation between the original sample and various subsamples as the sample size is systematically reduced. A CS‐C exceeding 0.25, 0.5, and 0.75 reflects acceptable, good, and excellent stability, respectively (Bringmann et al. 2019). Additionally, bootstrapped difference tests were performed to assess whether edge weights and node centralities differed significantly.

#### 2.3.3 Flow Network Structure

To further explore the relations among variables, we used the *flow* function to generate two flow diagrams visualizing the associations between FoMO, SNS addictive tendencies, nomophobia, and mental health problems—*anxiety* and *depression*, respectively (Epskamp et al. 2018, 2012). In each flow diagram, anxiety or depression was positioned on the left, allowing for the visualization of both direct and indirect connections with other variables. To rule out the influence of demographic factors, we estimated two flow networks with demographics as covariates (gender, age, only child, parental marriage status, and quality of parental marriage). We then conducted the Wilcoxon tests to compare the edge‐weight matrices of the flow networks with and without these covariates.

## 3 Results

### 3.1 Descriptive Statistics

**FIGURE 1:**
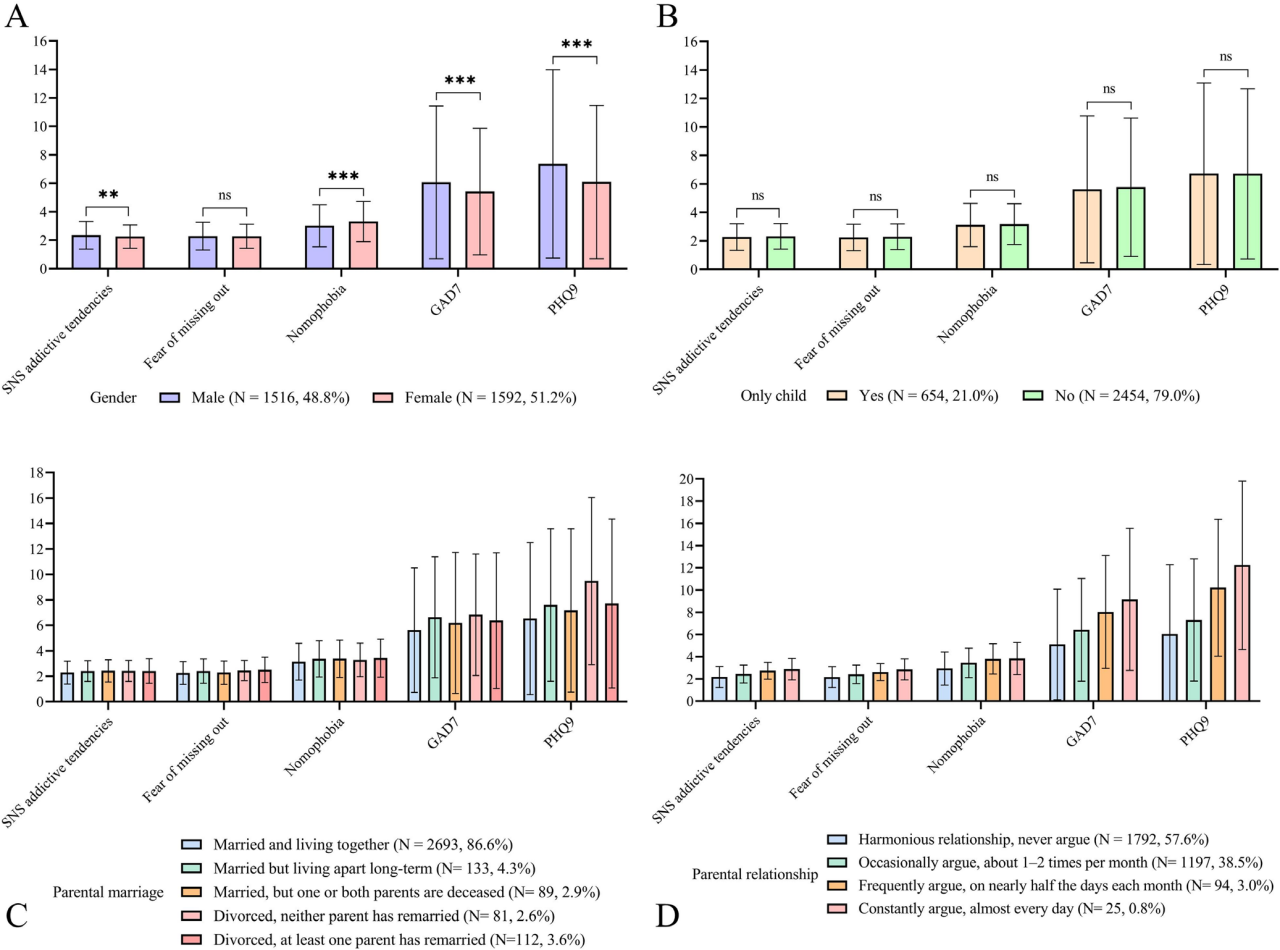
Descriptive statistics of variables grouped by demographic characteristics. See Table S1 for the correlations between demographic characteristics and target variables.

Figure 1 shows the descriptive statistics of target variables grouped by demographic characteristics. Male students reported higher levels of SNS addictive tendencies, anxiety, and depression (*p*s ≤ 0.006, Cohen's *d*s ≥ 0.10) but lower levels of nomophobia (*p* < 0.001, Cohen's *d* = −0.20; Figure 1A). No significant gender differences were found in FoMO (*p* = 0.895). Additionally, no significant differences were observed between only‐child and nononly‐child participants across all variables (*p*s ≥ 0.196; Figure 1B).

Table S1 displays the correlations between variables of interest and demographic information. As shown in parts (C) and (D) of Figure 1, poorer parental marital status (*r*s = 0.04–0.09, *p*s ≤ 0.019) and lower quality of parental marriage (*r*s = 0.16–0.18, *p*s ≤ 0.001) were associated with higher levels of all measured variables.

### 3.2 Network Structures and Centrality

**FIGURE 2:**
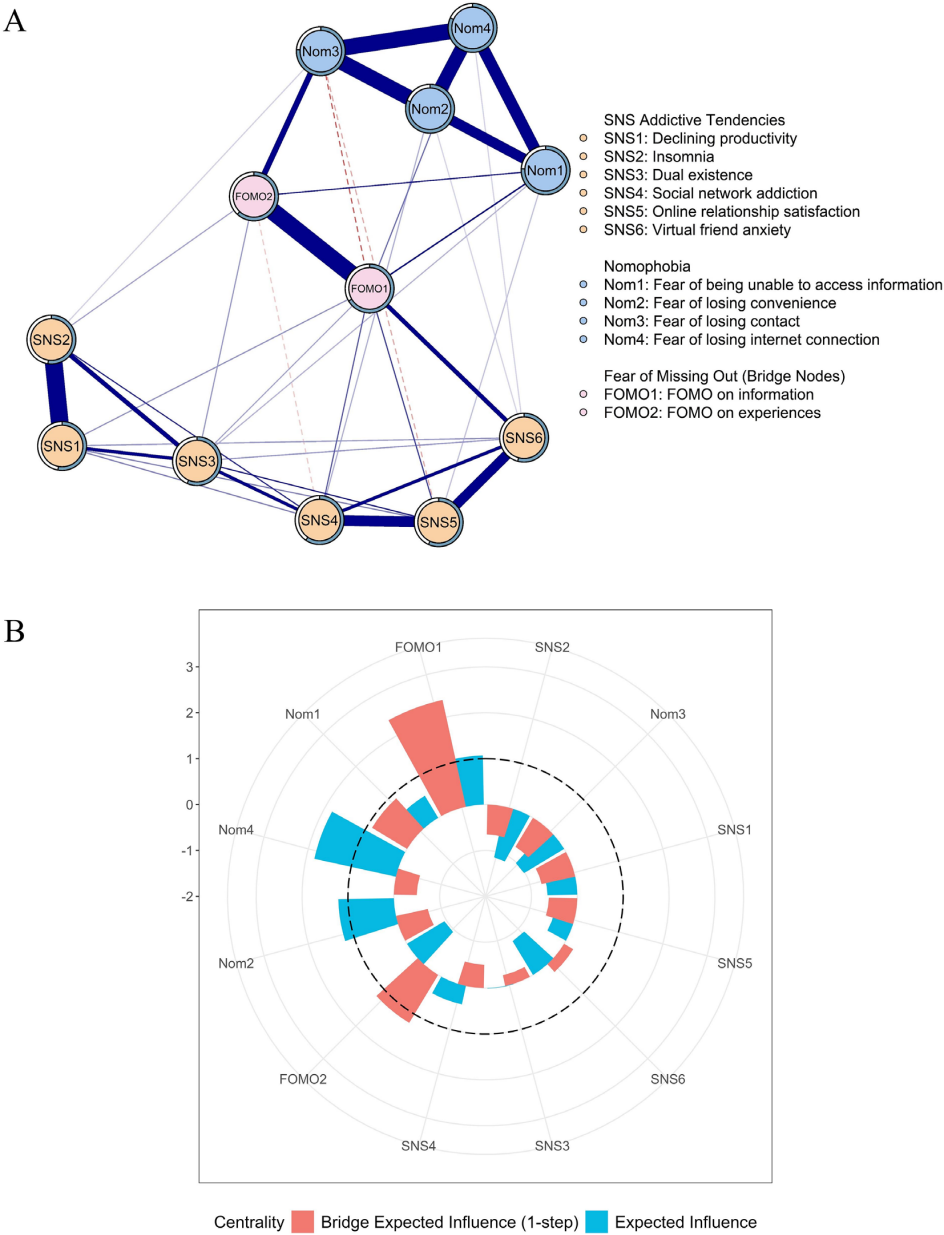
Network structures (A) and node centrality (B). In part (A), thicker lines represent stronger connections; blue solid lines indicate positive associations, while red dashed lines represent negative ones.

Figure 2A shows the network structures of the variables (see Table S2 for the full edge‐weight matrix). For the nomophobia community, “fear of losing internet connection” (Nom4; 0.305 ≤ *r* ≤ 0.364) and “fear of losing convenience” (Nom2; 0.284 ≤ *r* ≤ 0.360) displayed strong positive correlations with the other three factors of nomophobia, excluding themselves. For the FoMO community, “FoMO on information” (FoMO1) was positively correlated with “FoMO on the situation” (FoMO2; *r* = 0.387). In addition, the edge between “declining productivity” and “insomnia” (SNS1‐SNS2; *r* = 0.390) was the strongest edge within the SNS addictive tendencies community. For the whole network, FoMO1 showed strong positive associations with “virtual friend anxiety” (SNS6; *r* = 0.197), “online relationship satisfaction” (SNS5; *r* = 0.113), and “social network addiction” (SNS4; *r* = 0.098). In contrast, FoMO2 showed only weak positive connections with “dual existence” (SNS3; *r* = 0.060) and “insomnia” (SNS2; *r* = 0.056). In addition, both “FoMO on information” (FoMO1; *r* = 0.154) and “FoMO on situation” (FoMO2; *r* = 0.136) displayed positive associations with “fear of being unable to access information” (Nom1). Additionally, “FoMO on information” (FoMO1; *r* = 0.154) was also positively associated with “fear of losing internet connection” (Nom4; *r* = 0.086). Notably, “FoMO on information” (FoMO1; *r* = −0.117) and “FoMO on the situation” (FoMO2; *r* = 0.217) displayed adverse associations with “fear of losing contact” (Nom3).

“Fear of losing internet connection” (Nom4) had the highest EI value (Figure 2B and Table S2), followed by “fear of losing convenience” (Nom2) and “FoMO on information” (FoMO1), identifying these three nodes as the most central factors in the network. The two FoMO factors—“FoMO on information” (FoMO1) and “FoMO on the situation” (FoMO2)—had the highest bridge EI values (Figure 2B and Table S2), highlighting their roles as key bridge factors connecting to SNS addictive tendencies and nomophobia.

Additionally, we estimated an additional network, including demographic covariates such as gender, age, only child (y/n), parental marriage status, and quality of parental marriage (see Table S3 for the full edge‐weight matrix). We found no significant differences between the two matrices after comparing the edge‐weight matrices of networks with and without covariates (*p* = 0.779). Therefore, the estimated network was not influenced by demographics.

### 3.3 Network Accuracy and Stability

Before examining the network's accuracy and stability, we estimated an additional network, including demographic covariates such as gender, age, only child (y/n), parental marriage status, and quality of parental marriage. We found no significant differences between the two matrices after comparing the edge‐weight matrices of networks with and without covariates (*p* = 0.779). Therefore, the estimated network was not influenced by demographics.

The bootstrapped analysis for edge weights (Figure S1) showed that the 95% CIs for edge weights were adequately narrow, indicating the high accuracy of the estimated network. The case‐dropping bootstrap analyses for centrality (Figure S2) further indicated excellent stability for both EI (CS‐C = 0.75) and bridge EI (CS‐C = 0.75). In addition, nonparticipation tests revealed significant differences in both edge weights (Figure S3) and centrality indices (Figure S4; e.g., EI and bridge EI).

### 3.4 Flow Networks for Anxiety and Depression

**FIGURE 3:**
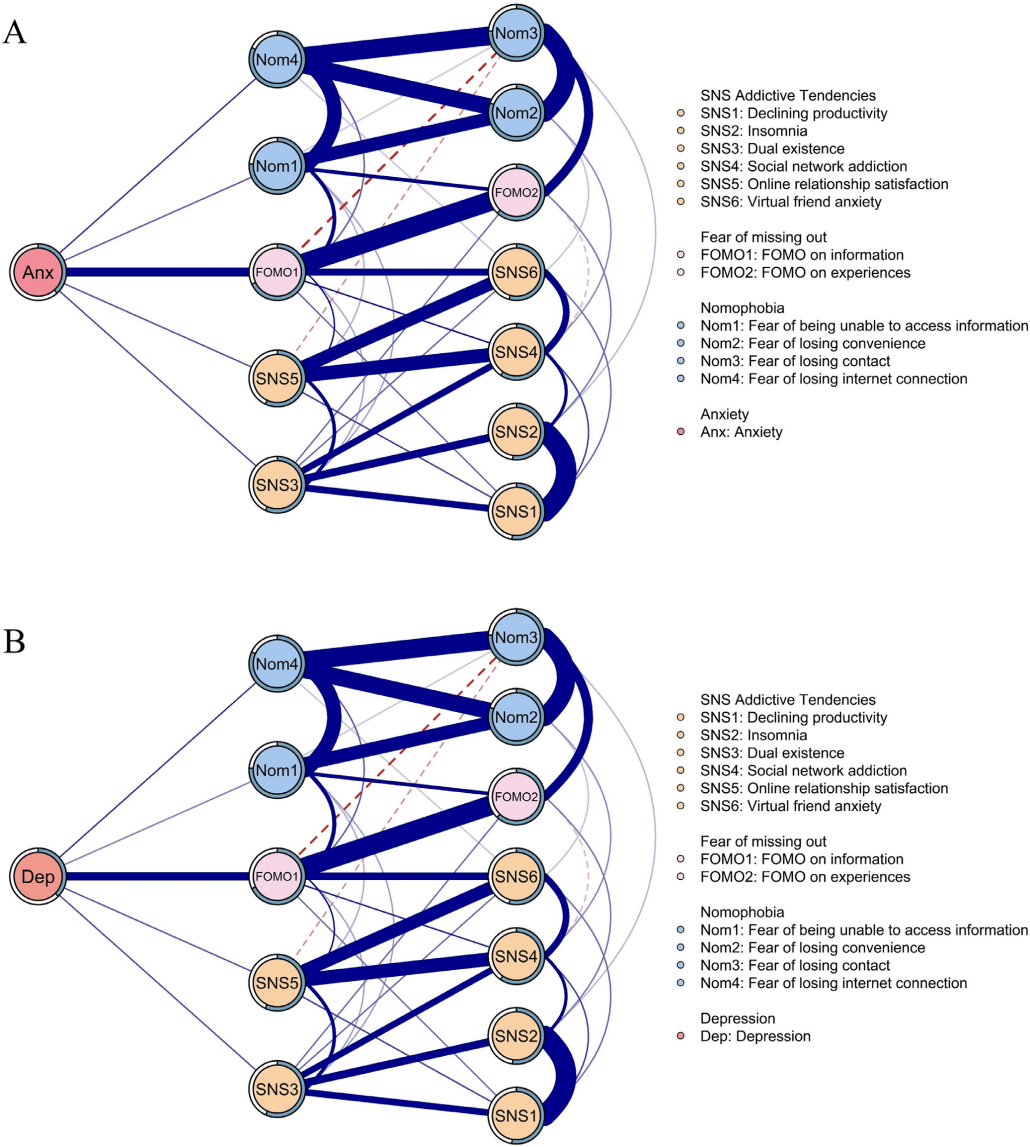
Flow networks of anxiety (A) and depression (B).

Figure 3 shows the flow networks for anxiety and depression (see Tables S4 and S5 for the edge‐weight matrices). Among all factors, “FoMO on information” (FoMO1) was the factor most highly associated with both anxiety (Anx; *r* = 0.214) and depression (Dep; *r* = 0.205). Other factors associated with anxiety included “fear of losing internet connection” (Nom4; *r* = 0.081), “dual existence” (SNS3; *r* = 0.073), “online relationship satisfaction” (SNS5; *r* = 0.069), and “fear of being unable to access information” (Nom1; *r* = 0.064). Similarly, these four factors were also linked to depression (Dep; Nom4, *r* = 0.082; SNS3, *r* = 0.071; SNS5, *r* = 0.066; Nom1, *r* = 0.050). The additional flow networks for anxiety and depression were then estimated with demographics as covariates (see Tables S6 and S7 for the full edge‐weight matrices). Wilcoxon tests showed no significant differences between the edge‐weight matrices of networks with and without covariates (Anxiety: *p* = 0.788; Depression: *p* = 0.777).

## 4 Discussion

The present study employed network analysis to elucidate the intricate relationships among symptoms of nomophobia, SNS addiction, and FoMO, identifying specific bridge symptoms that connect these behaviors to adverse mental health outcomes. Some findings deserve discussion.

Notably, the nomophobia symptom, characterized by the fear of being unable to communicate or access information, demonstrated the strongest direct association with anxiety symptoms. This finding aligns with prior research indicating that disconnection from digital communication, particularly among heavy smartphone users, can elicit acute anxiety responses (Cheever et al. 2021). Furthermore, FoMO, particularly the persistent concern about being excluded from social experiences, was identified as a significant predictor of depressive symptoms. This result is consistent with a growing body of literature suggesting that FoMO is positively associated with elevated levels of both depression and anxiety (Liu et al. 2023). Importantly, these findings suggest that not all facets of technology‐related behaviors confer equal psychological risk. Rather, specific maladaptive cognitive patterns, such as intrusive thoughts about being excluded or disconnected (e.g., “I'm missing something important”), may function as bridge mechanisms linking digital overdependence to psychological distress.

These findings lend support to an emerging theoretical framework suggesting that digital behavior‐related addictions and emotional disorders are interconnected through overlapping bridge symptoms. According to the network theory of psychopathology, mental disorders arise from direct and dynamic interactions among individual symptoms (Borsboom 2017). Within this framework, the symptom of “fear of disconnection” may serve as a critical node linking nomophobia and excessive social networking use with anxiety and depression, contributing to a self‐perpetuating cycle of psychological distress. Such bridge symptoms may help explain the frequent co‐occurrence of problematic smartphone use and mood disorders, as they facilitate the transmission of distress across otherwise distinct symptom clusters (Borsboom 2017). In line with this perspective, our results indicate that acute anxiety in response to disconnection may not only reflect pre‐existing vulnerability but could also activate or intensify broader patterns of anxious and depressive symptoms (Guo et al. 2025; Tao, Tang, et al. 2023; Tao, Wang, et al. 2023), especially in adolescents who have lower self‐control ability (Lu et al. 2025). This process may, in turn, reinforce reliance on digital devices, thereby sustaining a cycle of dependence and impaired mental health.

This study contributes to the understanding of how specific facets of nomophobia and related behaviors impact mental health. While excessive smartphone and social media use is linked to poorer mental health (Keles et al. 2020), our symptom‐level analysis reveals that it is the anxiety over disconnection, rather than the sheer volume of use, that is most harmful. This finding aligns with previous research showing that the fear of losing phone access can trigger measurable anxiety and stress (Cheever et al. 2021). Similarly, we found that FoMO is directly associated with depressive symptoms, echoing the well‐established link between FoMO and increased anxiety and depression, particularly among young people (Liu et al. 2023). We also observed gender differences, with female participants reporting higher levels of nomophobia and SNS addiction. This pattern is consistent with prior literature (e.g., Vagka et al. 2023) and has been widely observed in other studies, which often attribute women's higher nomophobia to a greater emotional attachment to smartphones and more intensive use of phones for social connectivity. Psychologically, female students may experience stronger anxiety about social separation or missing important communications, and sociocultural expectations might reinforce a need for women to remain constantly available and connected. Guo et al. (2025) have even noted that women tend to place a higher value on continuous social contact and may view their phones as vital for social support and safety, which could amplify anxiety when access is cut off. This suggests that women may indeed be more vulnerable to nomophobic anxiety due to their greater reliance on digital communication for social connection. To sum up, our results emphasize that the psychological impact of digital behaviors varies across individuals, and interventions should be tailored to account for gender, family context, and other demographic factors.

### 4.1 Theoretical and Practical Implications

Theoretically, our results underscore the value of a symptom‐specific, network‐based understanding of tech‐related mental health issues. Rather than viewing nomophobia, SNS addiction, and FoMO as isolated disorders or merely as summed scores on a questionnaire, it is more illuminating to see them as networks of interrelated symptoms that can spill into traditional mental disorders. By identifying *which* symptoms act as the critical connectors, we gain insight into the pathways through which, say, a benign habit of smartphone checking might evolve into clinical anxiety or depression. This perspective dovetails with contemporary network models of mental disorders, which argue that conditions like anxiety or depression emerge from interconnected symptom networks rather than a single latent cause (Borsboom 2017). Our study provides an example of this: the “nodes” of fear‐of‐disconnection and fear‐of‐missing‐out form bridges linking a network of digital‐behavior symptoms to a network of emotional distress symptoms. Such insight contributes to a more nuanced model of comorbidity in the digital age—one that can account for why problematic phone use, FoMO, and mood problems often coincide.

These insights also carry concrete practical implications. Interventions could be more effective if they target the specific problematic symptoms identified (e.g., the bridge symptom “FoMO on information” highlighted in our analysis), rather than addressing nomophobia or social media addiction in a broad sense. One promising approach is training individuals to cope with voluntary disconnection, which could directly alleviate the nomophobia‐related anxiety found to be particularly influential in this study. Such training can be grounded in cognitive‐behavioral therapy (CBT) principles, pairing gradual exposure to being offline with emotion regulation strategies and cognitive restructuring to challenge catastrophic thinking about disconnection. Therapeutic strategies like *exposure to short periods of being offline*, mindfulness techniques (as an emotion regulation strategy) to tolerate the discomfort of missing out, or cognitive restructuring to challenge catastrophic thoughts about not immediately responding to messages could help weaken the grip of those key symptoms. By reducing the central “fear of missing out” and “fear of no access” nodes, we might not only reduce those particular anxieties but also produce downstream improvements in general anxiety and depression. Our findings suggest a pinpoint approach: rather than broadly banning phones or apps (which is often unrealistic), helping individuals manage the emotional reactions tied to phone unavailability and social exclusion may yield better mental health outcomes.

Focusing on symptom‐level intervention aligns with personalized mental health care principles and may enhance intervention acceptability. For example, individuals can be trained to manage distress associated with delayed responses or temporary disconnection without requiring complete disengagement from technology. These findings also have practical implications for policymakers and educators, such as implementing digital literacy training workshops to foster healthier digital habits and critical thinking about online content among students. Such programs would equip young people with skills to manage information overload, potentially alleviating the irrational urgency to remain constantly connected that underlies symptoms like FoMO on information.

### 4.2 Limitations and Future Directions

Despite the strengths of our symptom network approach, several limitations warrant caution. First, our study is cross‐sectional, capturing a snapshot in time. We have interpreted the associations as certain symptoms affecting mental health, but it is equally plausible that individuals with higher anxiety or depression become more prone to nomophobic and FoMO fears. The relationship is likely bidirectional (Cheever et al. 2021), multicenter (Xu et al. 2025) and longitudinal studies are needed to untangle cause and effect. Future research should track people over time (e.g., observing if initial high FoMO predicts subsequent increases in depression, or vice versa) to confirm the directional influence of these symptom connections.

Second, the study relied exclusively on self‐report measures, which may introduce various biases. For instance, individuals with higher anxiety levels might overestimate their phone‐related fears, contributing to common method variance. Additionally, self‐reported data are inherently susceptible to recall inaccuracies and subjective interpretation, even when using validated instruments. To enhance measurement validity, future research should consider incorporating objective indicators, such as smartphone usage logs to capture actual checking behaviors, or physiological assessments (e.g., heart rate variability, skin conductance) during periods of disconnection to directly assess anxiety responses. Combining self‐report with behavioral and biological data would provide a more comprehensive understanding of nomophobia‐related distress. Moreover, experimental designs, randomly assigning participants to short‐term phone abstinence conditions, could help establish the temporal causal impact of disconnection on mood and anxiety symptoms with greater methodological rigor.

Third, our sample was comprised exclusively of Chinese college students, which may limit the generalizability of the findings to other cultural or demographic groups. Cultural factors and distinct digital environments could influence the prominence and impact of certain symptoms. For instance, “FoMO on information” might not emerge as strongly in populations with different social media use patterns. Therefore, caution is warranted in extending these results to non‐Chinese or older populations. Future research should include cross‐cultural comparisons and recruit more diverse samples (e.g., from other countries or age groups) to examine whether the identified network structure and bridge symptoms hold across different contexts.

Finally, while our network approach provides rich insight, it is inherently exploratory in nature. There is a risk of over‐interpreting connections that may not be robust. We addressed this by using established network estimation techniques and conducting stability checks (as described in the methods), but some reported edges could be sample‐specific. Replication is essential (Xu et al. 2025). Future studies should replicate our symptom network in other samples and ideally perform network comparison tests to formally assess differences (e.g., do males vs. females have different network structures? Do cultural groups differ in which symptom is most central?). Such work will refine the understanding of these interlinked phenomena and test the stability of the bridge symptoms we identified.

## 5 Conclusion

In summary, “FoMO on information” emerged as a core bridging symptom linking nomophobia and SNS addiction, representing a shared vulnerability that contributes to both anxiety and depression. This key finding highlights a clear target for intervention and illustrates the value of symptom‐level network analysis in addressing digital‐age mental health challenges.

## Funding

This work was supported by the 2025 Annual Project of the Tao Fen Foundation: Embodied Intelligence Empowering Nationwide Reading: Integrated Pathways of Technological Innovation and Reading Promotion (TF2025170).

## Ethics Statement

The authors assert that all procedures contributing to this work comply with the ethical standards of the relevant national and institutional committees on human experimentation and with the Helsinki Declaration of 1975, as revised in 2008. This research was examined and approved by the ethical committee of Peking University of Posts and Telecommunications (Reference number: BUPT‐P‐2025017).

## Conflicts of Interest

The authors declare no conflicts of interest.

## Supporting information


**Data S1:** pchj70068‐sup‐0001‐Supinfo.docx.

## Data Availability

The data that support the findings of this study are available from the corresponding author upon reasonable request.
