# Supplementary material for: Which Symptoms of Nomophobia, Social Networking Site Addiction, and Fear of Missing Out (FoMO) Directly Affect Mental Health? A Symptom Network and Flow Analysis Study
Source: Psych J. 2025 Nov 28;15(1):e70068. doi: 10.1002/pchj.70068 (PMC12856220; doi:10.1002/pchj.70068)
Supplement: Supplementary file 1 — Data S1: pchj70068‐sup‐0001‐Supinfo.docx. [file PCHJ-15-e70068-s001.docx]

**Online Supplemental Materials**

**Tables**

**Table S1: Descriptive Statistics and Correlation Matrix.**

| **Variable** | ***M*** | | ***SD*** | **1** | **2** | **3** | **4** | **5** | **6** | **7** | **8** | **9** |
| --- | --- | --- | --- | --- | --- | --- | --- | --- | --- | --- | --- | --- |
| 1. Gender | 1.51 |  | 0.50 | — |  |  |  |  |  |  |  |  |
| 2. Age | 20.75 |  | 1.61 | -.01 | — |  |  |  |  |  |  |  |
| 3. Only child | 1.79 |  | 0.41 | .13^***^ | .02 | — |  |  |  |  |  |  |
| 4. Parental marriage | 1.31 |  | 0.90 | .01 | -.03 | -.09^***^ | — |  |  |  |  |  |
| 5. Parental relationship | 1.47 |  | 0.60 | .14^***^ | -.04^*^ | .01 | .17^***^ | — |  |  |  |  |
| 6. SNS Addictive Tendencies (SNS) | 2.31 |  | 0.90 | -.05^**^ | -.05^**^ | .02 | .04^*^ | .18^***^ | — |  |  |  |
| 7. Fear of missing out (FOMO) | 2.28 |  | 0.91 | -.00 | -.13^***^ | .02 | .06^**^ | .16^***^ | .69^***^ | — |  |  |
| 8. Nomophobia | 3.17 |  | 1.45 | .10^***^ | -.11^***^ | .02 | .05^**^ | .19^***^ | .59^***^ | .72^***^ | — |  |
| 9. Anxiety (GAD7) | 5.74 |  | 4.92 | -.07^***^ | -.07^***^ | .01 | .06^**^ | .16^***^ | .51^***^ | .55^***^ | .51^***^ | — |
| 10. Depression (PHQ9) | 6.71 |  | 6.05 | -.11^***^ | -.06^***^ | -.00 | .09^***^ | .16^***^ | .52^***^ | .54^***^ | .49^***^ | .83^***^ |

*Note*. * *p* < .05. ** *p* < .01. *** *p* < .001.

**Table S2: The Edge-Weight Matrix and Standardized Centrality in the Network.**

| **Node** |  | **Edge-weight matrix** | | | | | | | | | | | |  | **Standardized Centrality** | | | |
| --- | --- | --- | --- | --- | --- | --- | --- | --- | --- | --- | --- | --- | --- | --- | --- | --- | --- | --- |
|  |  | **SNS1** | **SNS2** | **SNS3** | **SNS4** | **SNS5** | **SNS6** | **FOMO1** | **FOMO2** | **Nom1** | **Nom2** | **Nom3** | **Nom4** |  | **EI** | | **Bridge EI** | |
| **SNS** **Addictive Tendencies (SNS)** |  |  |  |  |  |  |  |  |  |  |  |  |  |  |  |  |  |  |
| SNS1: Declining productivity |  | 0 | 0.390 | 0.182 | 0.077 | 0.077 | 0.060 | 0.064 | 0 | 0 | 0 | 0 | 0 |  | -0.65 |  | -0.75 |  |
| SNS2: Insomnia |  | 0.390 | 0 | 0.200 | 0.127 | 0 | 0 | 0 | 0.056 | 0 | 0 | 0.027 | 0 |  | -1.13 |  | -0.65 |  |
| SNS3: Dual existence |  | 0.182 | 0.200 | 0 | 0.180 | 0.139 | 0.062 | 0.054 | 0.060 | 0.045 | 0 | 0 | 0 |  | 0.01 |  | -0.24 |  |
| SNS4: Social network addiction |  | 0.077 | 0.127 | 0.180 | 0 | 0.290 | 0.180 | 0.098 | -0.036 | 0 | 0.047 | 0 | 0 |  | 0.41 |  | -0.51 |  |
| SNS5: Online relationship satisfaction |  | 0.077 | 0 | 0.139 | 0.290 | 0 | 0.278 | 0.113 | 0 | 0.036 | 0 | -0.061 | 0 |  | -0.46 |  | -0.62 |  |
| SNS6: Virtual friend anxiety |  | 0.060 | 0 | 0.062 | 0.180 | 0.278 | 0 | 0.197 | 0 | 0 | 0.025 | 0 | 0.026 |  | -0.86 |  | 0.23 |  |
| **Fear of missing out (FOMO)** |  |  |  |  |  |  |  |  |  |  |  |  |  |  |  |  |  |  |
| FOMO1: FOMO on information |  | 0.064 | 0 | 0.054 | 0.098 | 0.113 | 0.197 | 0 | 0.387 | 0.154 | 0 | -0.117 | 0.086 |  | 1.07 |  | 2.38 |  |
| FOMO2: FOMO on experiences |  | 0 | 0.056 | 0.060 | -0.036 | 0 | 0 | 0.387 | 0 | 0.136 | 0 | 0.217 | 0 |  | -0.95 |  | 1.22 |  |
| **Nomophobia (Nom)** |  |  |  |  |  |  |  |  |  |  |  |  |  |  |  |  |  |  |
| Nom1: Fear of being unable to access information |  | 0 | 0 | 0.045 | 0 | 0.036 | 0 | 0.154 | 0.136 | 0 | 0.284 | 0.021 | 0.305 |  | 0.56 |  | 0.89 |  |
| Nom2: Fear of losing convenience |  | 0 | 0 | 0 | 0.047 | 0 | 0.025 | 0 | 0 | 0.284 | 0 | 0.360 | 0.334 |  | 1.21 |  | -0.71 |  |
| Nom3: Fear of losing contact |  | 0 | 0.027 | 0 | 0 | -0.061 | 0 | -0.117 | 0.217 | 0.021 | 0.360 | 0 | 0.364 |  | -1.02 |  | -0.74 |  |
| Nom4: Fear of losing internet connection |  | 0.000 | 0.000 | 0.000 | 0.000 | 0.000 | 0.026 | 0.086 | 0.000 | 0.305 | 0.334 | 0.364 | 0 |  | 1.82 |  | -0.50 |  |

***Note*.** EI, Expected Influence. Bridge EI, bridge Expected Influence.

**Table S3: The Edge-Weight Matrix of the Network with Demographics as Covariates.**

| **Node** |  | **Edge-weight matrix** | | | | | | | | | | | | | | | | |
| --- | --- | --- | --- | --- | --- | --- | --- | --- | --- | --- | --- | --- | --- | --- | --- | --- | --- | --- |
|  |  | **SNS1** | **SNS2** | **SNS3** | **SNS4** | **SNS5** | **SNS6** | **FOMO1** | **FOMO2** | **Nom1** | **Nom2** | **Nom3** | **Nom4** | **Gen** | **Age** | **OK** | **PM** | **PR** |
| **SNS Addictive Tendencies (SNS)** |  |  |  |  |  |  |  |  |  |  |  |  |  |  |  |  |  |  |
| SNS1: Declining productivity |  | 0 | 0.390 | 0.181 | 0.077 | 0.074 | 0.060 | 0.064 | 0 | 0 | 0 | 0 | 0 | 0 | 0 | 0 | 0 | 0 |
| SNS2: Insomnia |  | 0.390 | 0 | 0.199 | 0.125 | 0 | 0 | 0 | 0.056 | 0 | 0 | 0 | 0 | 0 | 0 | 0 | 0 | 0.062 |
| SNS3: Dual existence |  | 0.181 | 0.199 | 0 | 0.180 | 0.136 | 0.063 | 0.053 | 0.060 | 0.044 | 0 | 0 | 0 | 0 | 0 | 0 | 0 | 0 |
| SNS4: Social network addiction |  | 0.077 | 0.125 | 0.180 | 0 | 0.289 | 0.180 | 0.098 | 0 | 0 | 0.049 | 0 | 0 | 0 | 0 | 0 | 0 | 0 |
| SNS5: Online relationship satisfaction |  | 0.074 | 0 | 0.136 | 0.289 | 0 | 0.280 | 0.110 | 0 | 0.034 | 0 | -0.049 | 0 | -0.093 | 0 | 0 | 0 | 0 |
| SNS6: Virtual friend anxiety |  | 0.060 | 0 | 0.063 | 0.180 | 0.280 | 0 | 0.198 | 0 | 0 | 0 | 0 | 0 | 0 | 0 | 0 | 0 | 0 |
| **Fear of missing out (FOMO)** |  |  |  |  |  |  |  |  |  |  |  |  |  |  |  |  |  |  |
| FOMO1: FOMO on information |  | 0.064 | 0 | 0.053 | 0.098 | 0.110 | 0.198 | 0 | 0.384 | 0.153 | 0 | -0.114 | 0.084 | 0 | 0 | 0 | 0 | 0 |
| FOMO2: FOMO on experiences |  | 0 | 0.056 | 0.060 | 0 | 0 | 0 | 0.384 | 0 | 0.135 | 0 | 0.215 | 0 | 0 | 0 | 0 | 0 | 0 |
| **Nomophobia (Nom)** |  |  |  |  |  |  |  |  |  |  |  |  |  |  |  |  |  |  |
| Nom1: Fear of being unable to access information |  | 0 | 0 | 0.044 | 0 | 0.034 | 0 | 0.153 | 0.135 | 0 | 0.284 | 0.025 | 0.303 | 0 | 0 | 0 | 0 | 0 |
| Nom2: Fear of losing convenience |  | 0 | 0 | 0 | 0.049 | 0 | 0 | 0 | 0 | 0.284 | 0 | 0.351 | 0.334 | 0.055 | 0 | 0 | 0 | 0 |
| Nom3: Fear of losing contact |  | 0 | 0 | 0 | 0 | -0.049 | 0 | -0.114 | 0.215 | 0.025 | 0.351 | 0 | 0.365 | 0.098 | 0 | 0 | 0 | 0 |
| Nom4: Fear of losing internet connection |  | 0 | 0 | 0 | 0 | 0 | 0 | 0.084 | 0 | 0.303 | 0.334 | 0.365 | 0 | 0 | 0 | 0 | 0 | 0 |
| **Demographics** |  |  |  |  |  |  |  |  |  |  |  |  |  |  |  |  |  |  |
| Gen: Gender |  | 0 | 0 | 0 | 0 | -0.093 | 0 | 0 | 0 | 0 | 0.055 | 0.098 | 0 | 0 | 0 | 0.120 | 0 | 0.123 |
| Age: Age |  | 0 | 0 | 0 | 0 | 0 | 0 | 0 | 0 | 0 | 0 | 0 | 0 | 0 | 0 | 0 | 0 | 0 |
| OK: Only Kids (y/n) |  | 0 | 0 | 0 | 0 | 0 | 0 | 0 | 0 | 0 | 0 | 0 | 0 | 0.120 | 0 | 0 | 0 | 0 |
| PM: Parental marriage |  | 0 | 0 | 0 | 0 | 0 | 0 | 0 | 0 | 0 | 0 | 0 | 0 | 0 | 0 | 0 | 0 | 0.156 |
| PR: Parental relationship |  | 0 | 0.062 | 0 | 0 | 0 | 0 | 0 | 0 | 0 | 0 | 0 | 0 | 0.123 | 0 | 0 | 0.156 | 0 |

***Note*.** The Wilcoxon tests showed no significant differences between the edge-weight matrices of the networks with and without covariates (*p* = 0.779), suggesting minimal influence of demographic factors.

**Table S4: The Edge-Weight Matrix in the Flow Network for Anxiety.**

| **Node** |  | **Edge-weight matrix** | | | | | | | | | | | | |
| --- | --- | --- | --- | --- | --- | --- | --- | --- | --- | --- | --- | --- | --- | --- |
|  |  | **SNS1** | **SNS2** | **SNS3** | **SNS4** | **SNS5** | **SNS6** | **FOMO1** | **FOMO2** | **Nom1** | **Nom2** | **Nom3** | **Nom4** | **Anx** |
| **SNS Addictive Tendencies (SNS)** |  |  |  |  |  |  |  |  |  |  |  |  |  |  |
| SNS1: Declining productivity |  | 0 | 0.390 | 0.181 | 0.077 | 0.077 | 0.060 | 0.062 | 0 | 0 | 0 | 0 | 0 | 0 |
| SNS2: Insomnia |  | 0.390 | 0 | 0.199 | 0.127 | 0 | 0 | 0 | 0.056 | 0 | 0 | 0.028 | 0 | 0 |
| SNS3: Dual existence |  | 0.181 | 0.199 | 0 | 0.180 | 0.133 | 0.061 | 0.036 | 0.060 | 0.037 | 0 | 0 | 0 | 0.073 |
| SNS4: Social network addiction |  | 0.077 | 0.127 | 0.180 | 0 | 0.290 | 0.181 | 0.095 | -0.036 | 0 | 0.047 | 0 | 0 | 0 |
| SNS5: Online relationship satisfaction |  | 0.077 | 0 | 0.133 | 0.290 | 0 | 0.276 | 0.096 | 0 | 0.031 | 0 | -0.060 | 0 | 0.069 |
| SNS6: Virtual friend anxiety |  | 0.060 | 0 | 0.061 | 0.181 | 0.276 | 0 | 0.189 | 0 | 0 | 0.025 | 0 | 0.024 | 0 |
| **Fear of missing out (FOMO)** |  |  |  |  |  |  |  |  |  |  |  |  |  |  |
| FOMO1: FOMO on information |  | 0.062 | 0 | 0.036 | 0.095 | 0.096 | 0.189 | 0 | 0.378 | 0.137 | 0 | -0.112 | 0.066 | 0.214 |
| FOMO2: FOMO on experiences |  | 0 | 0.056 | 0.060 | -0.036 | 0 | 0 | 0.378 | 0 | 0.135 | 0 | 0.217 | 0 | 0 |
| **Nomophobia (Nom)** |  |  |  |  |  |  |  |  |  |  |  |  |  |  |
| Nom1: Fear of being unable to access information |  | 0 | 0 | 0.037 | 0 | 0.031 | 0 | 0.137 | 0.135 | 0 | 0.282 | 0.022 | 0.298 | 0.064 |
| Nom2: Fear of losing convenience |  | 0 | 0 | 0 | 0.047 | 0 | 0.025 | 0 | 0 | 0.282 | 0 | 0.360 | 0.331 | 0 |
| Nom3: Fear of losing contact |  | 0 | 0.028 | 0 | 0 | -0.060 | 0 | -0.112 | 0.217 | 0.022 | 0.360 | 0 | 0.364 | 0 |
| Nom4: Fear of losing internet connection |  | 0 | 0 | 0 | 0 | 0 | 0.024 | 0.066 | 0 | 0.298 | 0.331 | 0.364 | 0 | 0.081 |
| **Anxiety (Anx)** |  |  |  |  |  |  |  |  |  |  |  |  |  |  |
| Anx: Anxiety |  | 0 | 0 | 0.073 | 0 | 0.069 | 0 | 0.214 | 0 | 0.064 | 0 | 0 | 0.081 | 0 |

**Table S5: The Edge-Weight Matrix in the Flow Network for Depression.**

| **Node** |  | **Edge-weight matrix** | | | | | | | | | | | | |
| --- | --- | --- | --- | --- | --- | --- | --- | --- | --- | --- | --- | --- | --- | --- |
|  |  | **SNS1** | **SNS2** | **SNS3** | **SNS4** | **SNS5** | **SNS6** | **FOMO1** | **FOMO2** | **Nom1** | **Nom2** | **Nom3** | **Nom4** | **Dep** |
| **SNS Addictive Tendencies (SNS)** |  |  |  |  |  |  |  |  |  |  |  |  |  |  |
| SNS1: Declining productivity |  | 0 | 0.390 | 0.180 | 0.076 | 0.076 | 0.060 | 0.058 | 0 | 0 | 0 | 0 | 0 | 0 |
| SNS2: Insomnia |  | 0.390 | 0 | 0.199 | 0.127 | 0 | 0 | 0 | 0.056 | 0 | 0 | 0.028 | 0 | 0 |
| SNS3: Dual existence |  | 0.180 | 0.199 | 0 | 0.177 | 0.133 | 0.061 | 0.037 | 0.060 | 0.039 | 0 | 0 | 0 | 0.071 |
| SNS4: Social network addiction |  | 0.076 | 0.127 | 0.177 | 0 | 0.287 | 0.180 | 0.088 | -0.036 | 0 | 0.046 | 0 | 0 | 0 |
| SNS5: Online relationship satisfaction |  | 0.076 | 0 | 0.133 | 0.287 | 0 | 0.276 | 0.097 | 0 | 0.033 | 0 | -0.059 | 0 | 0.066 |
| SNS6: Virtual friend anxiety |  | 0.060 | 0 | 0.061 | 0.180 | 0.276 | 0 | 0.189 | 0 | 0 | 0.025 | 0 | 0.024 | 0 |
| **Fear of missing out (FOMO)** |  |  |  |  |  |  |  |  |  |  |  |  |  |  |
| FOMO1: FOMO on information |  | 0.058 | 0 | 0.037 | 0.088 | 0.097 | 0.189 | 0 | 0.378 | 0.141 | 0 | -0.109 | 0.067 | 0.205 |
| FOMO2: FOMO on experiences |  | 0 | 0.056 | 0.060 | -0.036 | 0 | 0 | 0.378 | 0 | 0.135 | 0 | 0.217 | 0 | 0 |
| **Nomophobia (Nom)** |  |  |  |  |  |  |  |  |  |  |  |  |  |  |
| Nom1: Fear of being unable to access information |  | 0 | 0 | 0.039 | 0 | 0.033 | 0 | 0.141 | 0.135 | 0 | 0.283 | 0.023 | 0.299 | 0.050 |
| Nom2: Fear of losing convenience |  | 0 | 0 | 0 | 0.046 | 0 | 0.025 | 0 | 0 | 0.283 | 0 | 0.360 | 0.332 | 0 |
| Nom3: Fear of losing contact |  | 0 | 0.028 | 0 | 0 | -0.059 | 0 | -0.109 | 0.217 | 0.023 | 0.360 | 0 | 0.365 | 0 |
| Nom4: Fear of losing internet connection |  | 0 | 0 | 0 | 0 | 0 | 0.024 | 0.067 | 0 | 0.299 | 0.332 | 0.365 | 0 | 0.082 |
| **Depression (Dep)** |  |  |  |  |  |  |  |  |  |  |  |  |  |  |
| Dep: Depression |  | 0 | 0 | 0.071 | 0 | 0.066 | 0 | 0.205 | 0 | 0.050 | 0 | 0 | 0.082 | 0 |

**Table S6: The Edge-Weight Matrix of the Flow Network** **for Anxiety with Demographics as Covariates.**

| **Node** |  |  | | **Edge-weight matrix** | | | | | | | | | | | | | | | | |
| --- | --- | --- | --- | --- | --- | --- | --- | --- | --- | --- | --- | --- | --- | --- | --- | --- | --- | --- | --- | --- |
|  |  | **SNS1** | **SNS2** | | **SNS3** | **SNS4** | **SNS5** | **SNS6** | **FOMO1** | **FOMO2** | **Nom1** | **Nom2** | **Nom3** | **Nom4** | Anx | **Gen** | **Age** | **OK** | **PM** | **PR** |
| **SNS Addictive Tendencies (SNS)** |  |  |  | |  |  |  |  |  |  |  |  |  |  |  |  |  |  |  |  |
| SNS1: Declining productivity |  | 0 | 0.390 | | 0.179 | 0.076 | 0.072 | 0.060 | 0.059 | 0 | 0 | 0 | 0 | 0 | 0 | 0 | 0 | 0 | 0 | 0 |
| SNS2: Insomnia |  | 0.390 | 0 | | 0.198 | 0.125 | 0 | 0 | 0 | 0.056 | 0 | 0 | 0 | 0 | 0 | 0 | 0 | 0 | 0 | 0.061 |
| SNS3: Dual existence |  | 0.179 | 0.198 | | 0 | 0.177 | 0.131 | 0.061 | 0.038 | 0.059 | 0.038 | 0 | 0 | 0 | 0.068 | 0 | 0 | 0 | 0 | 0 |
| SNS4: Social network addiction |  | 0.076 | 0.125 | | 0.177 | 0 | 0.286 | 0.179 | 0.089 | 0 | 0 | 0.049 | 0 | 0 | 0 | 0 | 0 | 0 | 0 | 0 |
| SNS5: Online relationship satisfaction |  | 0.072 | 0 | | 0.131 | 0.286 | 0 | 0.278 | 0.097 | 0 | 0.032 | 0 | -0.047 | 0 | 0.055 | -0.085 | 0 | 0 | 0 | 0 |
| SNS6: Virtual friend anxiety |  | 0.060 | 0 | | 0.061 | 0.179 | 0.278 | 0 | 0.189 | 0 | 0 | 0 | 0 | 0 | 0 | 0 | 0 | 0 | 0 | 0 |
| **Fear of missing out (FOMO)** |  |  |  | |  |  |  |  |  |  |  |  |  |  |  |  |  |  |  |  |
| FOMO1: FOMO on information |  | 0.059 | 0 | | 0.038 | 0.089 | 0.097 | 0.189 | 0 | 0.376 | 0.140 | 0 | -0.108 | 0.066 | 0.202 | 0 | 0 | 0 | 0 | 0 |
| FOMO2: FOMO on experiences |  | 0 | 0.056 | | 0.059 | 0 | 0 | 0 | 0.376 | 0 | 0.135 | 0 | 0.215 | 0 | 0 | 0 | 0 | 0 | 0 | 0 |
| **Nomophobia (Nom)** |  |  |  | |  |  |  |  |  |  |  |  |  |  |  |  |  |  |  |  |
| Nom1: Fear of being unable to access information |  | 0 | 0 | | 0.038 | 0 | 0.032 | 0 | 0.140 | 0.135 | 0 | 0.284 | 0.026 | 0.298 | 0.045 | 0 | 0 | 0 | 0 | 0 |
| Nom2: Fear of losing convenience |  | 0 | 0 | | 0 | 0.049 | 0 | 0 | 0 | 0 | 0.284 | 0 | 0.351 | 0.332 | 0 | 0.057 | 0 | 0 | 0 | 0 |
| Nom3: Fear of losing contact |  | 0 | 0 | | 0 | 0 | -0.047 | 0 | -0.108 | 0.215 | 0.026 | 0.351 | 0 | 0.365 | 0 | 0.096 | 0 | 0 | 0 | 0 |
| Nom4: Fear of losing internet connection |  | 0 | 0 | | 0 | 0 | 0 | 0 | 0.066 | 0 | 0.298 | 0.332 | 0.365 | 0 | 0.079 | 0 | 0 | 0 | 0 | 0 |
| **Anxiety (Anx)** |  |  |  | |  |  |  |  |  |  |  |  |  |  |  |  |  |  |  |  |
| Anx: Anxiety |  | 0 | 0 | | 0.068 | 0 | 0.055 | 0 | 0.202 | 0 | 0.045 | 0 | 0 | 0.079 | 0 | -0.119 | 0 | 0 | 0 | 0 |
| **Demographics** |  |  |  | |  |  |  |  |  |  |  |  |  |  |  |  |  |  |  |  |
| Gen: Gender |  | 0 | 0 | | 0 | 0 | -0.085 | 0 | 0 | 0 | 0 | 0.057 | 0.096 | 0 | -0.119 | 0 | 0 | 0.119 | 0 | 0.130 |
| Age: Age |  | 0 | 0 | | 0 | 0 | 0 | 0 | 0 | 0 | 0 | 0 | 0 | 0 | 0 | 0 | 0 | 0 | 0 | 0 |
| OK: Only Kids (y/n) |  | 0 | 0 | | 0 | 0 | 0 | 0 | 0 | 0 | 0 | 0 | 0 | 0 | 0 | 0.119 | 0 | 0 | 0 | 0 |
| PM: Parental marriage |  | 0 | 0 | | 0 | 0 | 0 | 0 | 0 | 0 | 0 | 0 | 0 | 0 | 0 | 0 | 0 | 0 | 0 | 0.152 |
| PR: Parental relationship |  | 0 | 0.061 | | 0 | 0 | 0 | 0 | 0 | 0 | 0 | 0 | 0 | 0 | 0 | 0.130 | 0 | 0 | 0.152 | 0 |

***Note*.** The Wilcoxon tests showed no significant differences between the edge-weight matrices of the networks with and without covariates (*p* = .777), suggesting minimal influence of demographic factors.

**Table S7: The Edge-Weight Matrix of the Flow Network** **for Depression with Demographics as Covariates.**

| **Node** |  |  | | **Edge-weight matrix** | | | | | | | | | | | | | | | | |
| --- | --- | --- | --- | --- | --- | --- | --- | --- | --- | --- | --- | --- | --- | --- | --- | --- | --- | --- | --- | --- |
|  |  | **SNS1** | **SNS2** | | **SNS3** | **SNS4** | **SNS5** | **SNS6** | **FOMO1** | **FOMO2** | **Nom1** | **Nom2** | **Nom3** | **Nom4** | Anx | **Gen** | **Age** | **OK** | **PM** | **PR** |
| **SNS Addictive Tendencies (SNS)** |  |  |  | |  |  |  |  |  |  |  |  |  |  |  |  |  |  |  |  |
| SNS1: Declining productivity |  | 0 | 0.390 | | 0.180 | 0.077 | 0.073 | 0.060 | 0.062 | 0 | 0 | 0 | 0 | 0 | 0 | 0 | 0 | 0 | 0 | 0 |
| SNS2: Insomnia |  | 0.390 | 0 | | 0.198 | 0.125 | 0 | 0 | 0 | 0.056 | 0 | 0 | 0 | 0 | 0 | 0 | 0 | 0 | 0 | 0.061 |
| SNS3: Dual existence |  | 0.180 | 0.198 | | 0 | 0.180 | 0.131 | 0.061 | 0.036 | 0.059 | 0.037 | 0 | 0 | 0 | 0.070 | 0 | 0 | 0 | 0 | 0 |
| SNS4: Social network addiction |  | 0.077 | 0.125 | | 0.180 | 0 | 0.289 | 0.180 | 0.096 | 0 | 0 | 0.049 | 0 | 0 | 0 | 0 | 0 | 0 | 0 | 0 |
| SNS5: Online relationship satisfaction |  | 0.073 | 0 | | 0.131 | 0.289 | 0 | 0.278 | 0.095 | 0 | 0.030 | 0 | -0.048 | 0 | 0.062 | -0.087 | 0 | 0 | 0 | 0 |
| SNS6: Virtual friend anxiety |  | 0.060 | 0 | | 0.061 | 0.180 | 0.278 | 0 | 0.189 | 0 | 0 | 0 | 0 | 0 | 0 | 0 | 0 | 0 | 0 | 0 |
| **Fear of missing out (FOMO)** |  |  |  | |  |  |  |  |  |  |  |  |  |  |  |  |  |  |  |  |
| FOMO1: FOMO on information |  | 0.062 | 0 | | 0.036 | 0.096 | 0.095 | 0.189 | 0 | 0.375 | 0.136 | 0 | -0.110 | 0.065 | 0.211 | 0 | 0 | 0 | 0 | 0 |
| FOMO2: FOMO on experiences |  | 0 | 0.056 | | 0.059 | 0 | 0 | 0 | 0.375 | 0 | 0.135 | 0 | 0.215 | 0 | 0 | 0 | 0 | 0 | 0 | 0 |
| **Nomophobia (Nom)** |  |  |  | |  |  |  |  |  |  |  |  |  |  |  |  |  |  |  |  |
| Nom1: Fear of being unable to access information |  | 0 | 0 | | 0.037 | 0 | 0.030 | 0 | 0.136 | 0.135 | 0 | 0.283 | 0.025 | 0.297 | 0.061 | 0 | 0 | 0 | 0 | 0 |
| Nom2: Fear of losing convenience |  | 0 | 0 | | 0 | 0.049 | 0 | 0 | 0 | 0 | 0.283 | 0 | 0.351 | 0.332 | 0 | 0.057 | 0 | 0 | 0 | 0 |
| Nom3: Fear of losing contact |  | 0 | 0 | | 0 | 0 | -0.048 | 0 | -0.110 | 0.215 | 0.025 | 0.351 | 0 | 0.364 | 0 | 0.098 | 0 | 0 | 0 | 0 |
| Nom4: Fear of losing internet connection |  | 0 | 0 | | 0 | 0 | 0 | 0 | 0.065 | 0 | 0.297 | 0.332 | 0.364 | 0 | 0.079 | 0 | 0 | 0 | 0 | 0 |
| **Anxiety (Anx)** |  |  |  | |  |  |  |  |  |  |  |  |  |  |  |  |  |  |  |  |
| Anx: Anxiety |  | 0 | 0 | | 0.070 | 0 | 0.062 | 0 | 0.211 | 0 | 0.061 | 0 | 0 | 0.079 | 0 | -0.080 | 0 | 0 | 0 | 0 |
| **Demographics** |  |  |  | |  |  |  |  |  |  |  |  |  |  |  |  |  |  |  |  |
| Gen: Gender |  | 0 | 0 | | 0 | 0 | -0.087 | 0 | 0 | 0 | 0 | 0.057 | 0.098 | 0 | -0.080 | 0 | 0 | 0.120 | 0 | 0.128 |
| Age: Age |  | 0 | 0 | | 0 | 0 | 0 | 0 | 0 | 0 | 0 | 0 | 0 | 0 | 0 | 0 | 0 | 0 | 0 | 0 |
| OK: Only Kids (y/n) |  | 0 | 0 | | 0 | 0 | 0 | 0 | 0 | 0 | 0 | 0 | 0 | 0 | 0 | 0.120 | 0 | 0 | 0 | 0 |
| PM: Parental marriage |  | 0 | 0 | | 0 | 0 | 0 | 0 | 0 | 0 | 0 | 0 | 0 | 0 | 0 | 0 | 0 | 0 | 0 | 0.155 |
| PR: Parental relationship |  | 0 | 0.061 | | 0 | 0 | 0 | 0 | 0 | 0 | 0 | 0 | 0 | 0 | 0 | 0.128 | 0 | 0 | 0.155 | 0 |

***Note*.** The Wilcoxon tests showed no significant differences between the edge-weight matrices of the networks with and without covariates (*p* = .788), suggesting minimal influence of demographic factors.

**Figures**


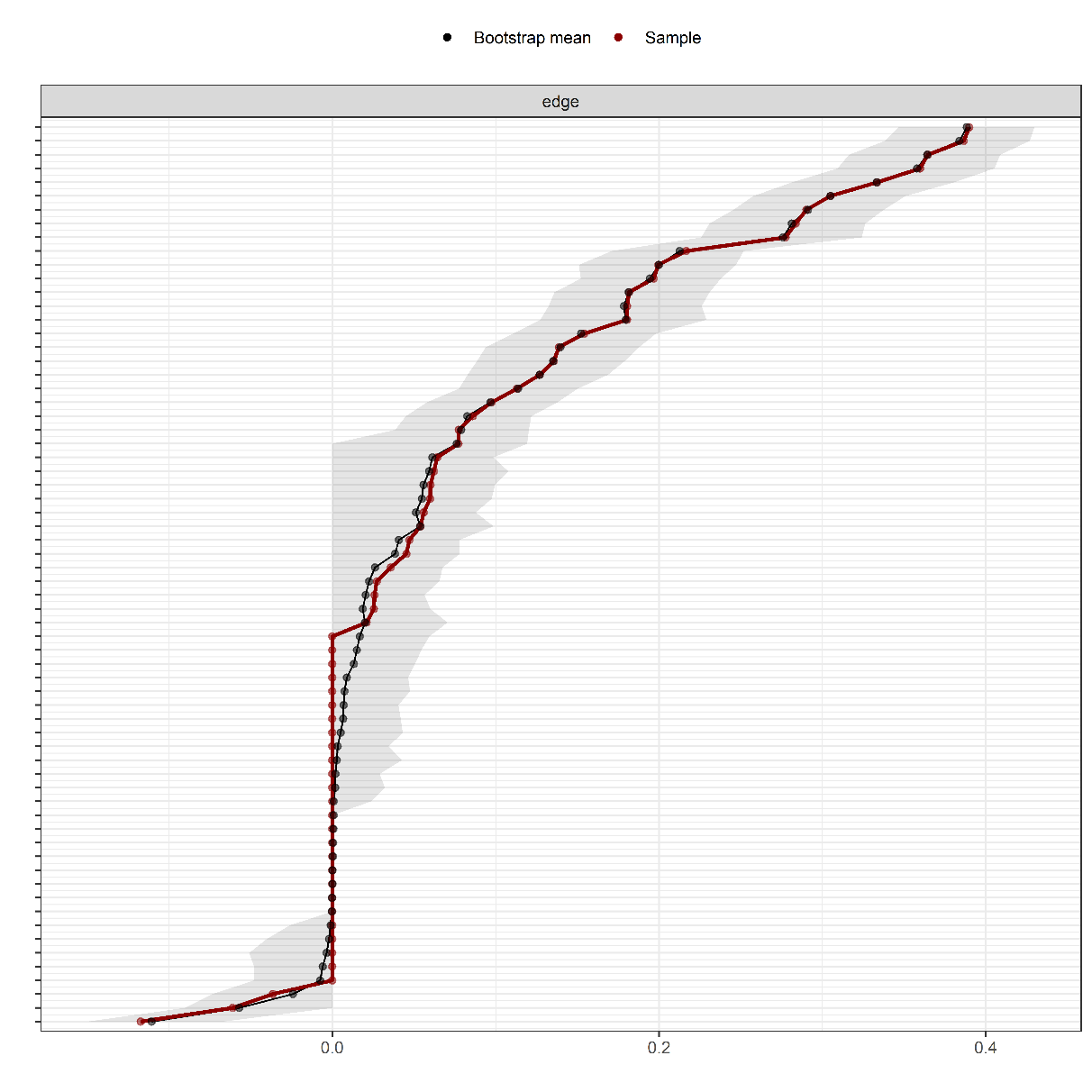


**Figure S1: Accuracy of edge weights for the network.**

***Note*.** the red line indicates the estimated edge, and the dark area indicates the 95% bootstrap CI.


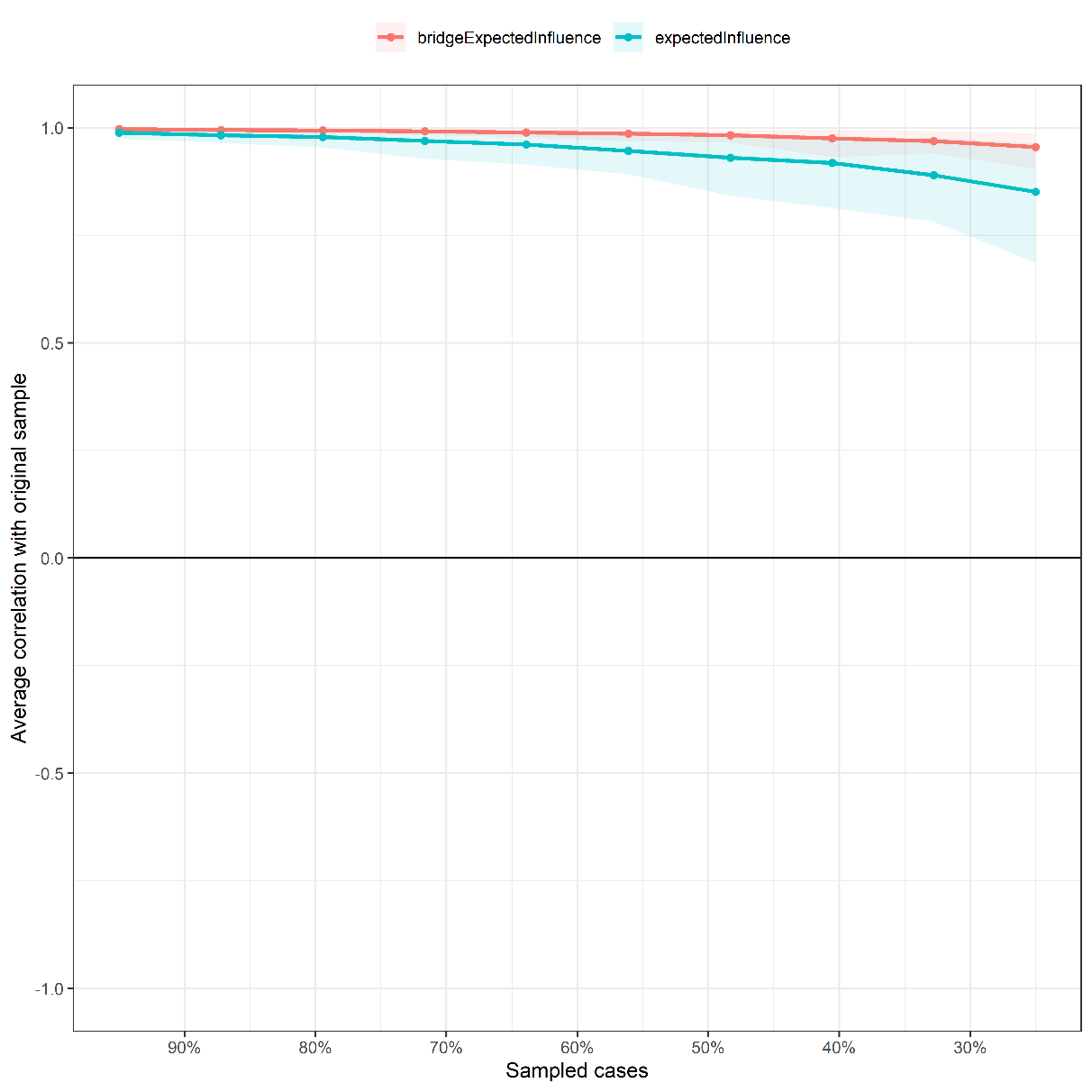


**Figure S2: Stability of EI and Bridge EI for the network.**

***Note*.** **y-axis**: Mean correlation between centrality metrics from the original network and those from networks re-estimated after progressively removing larger proportions of the sample. Correlation exceeding 0.25, 0.5, and 0.75 reflects acceptable, good, and excellent stability, respectively.


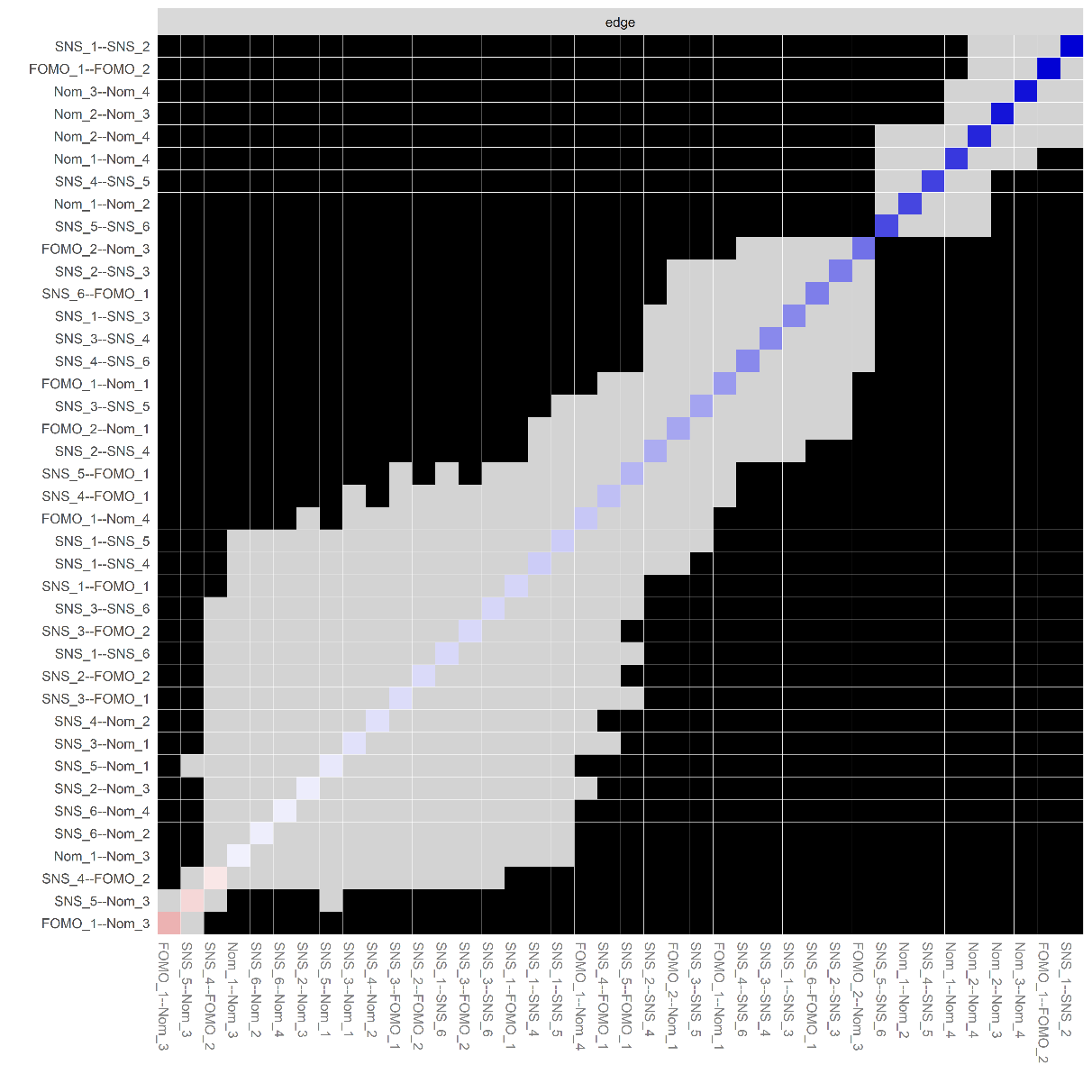


**Figure S3: Bootstrapped difference test for edge weights and in the network.**

***Note*.** Box colors indicate whether edge weights differ significantly: black denotes significant differences, while gray indicates no significant difference. The diagonal line reflects edge strength, with colors ranging from red (negative associations) to white (weaker edges) to blue (stronger positive connections).


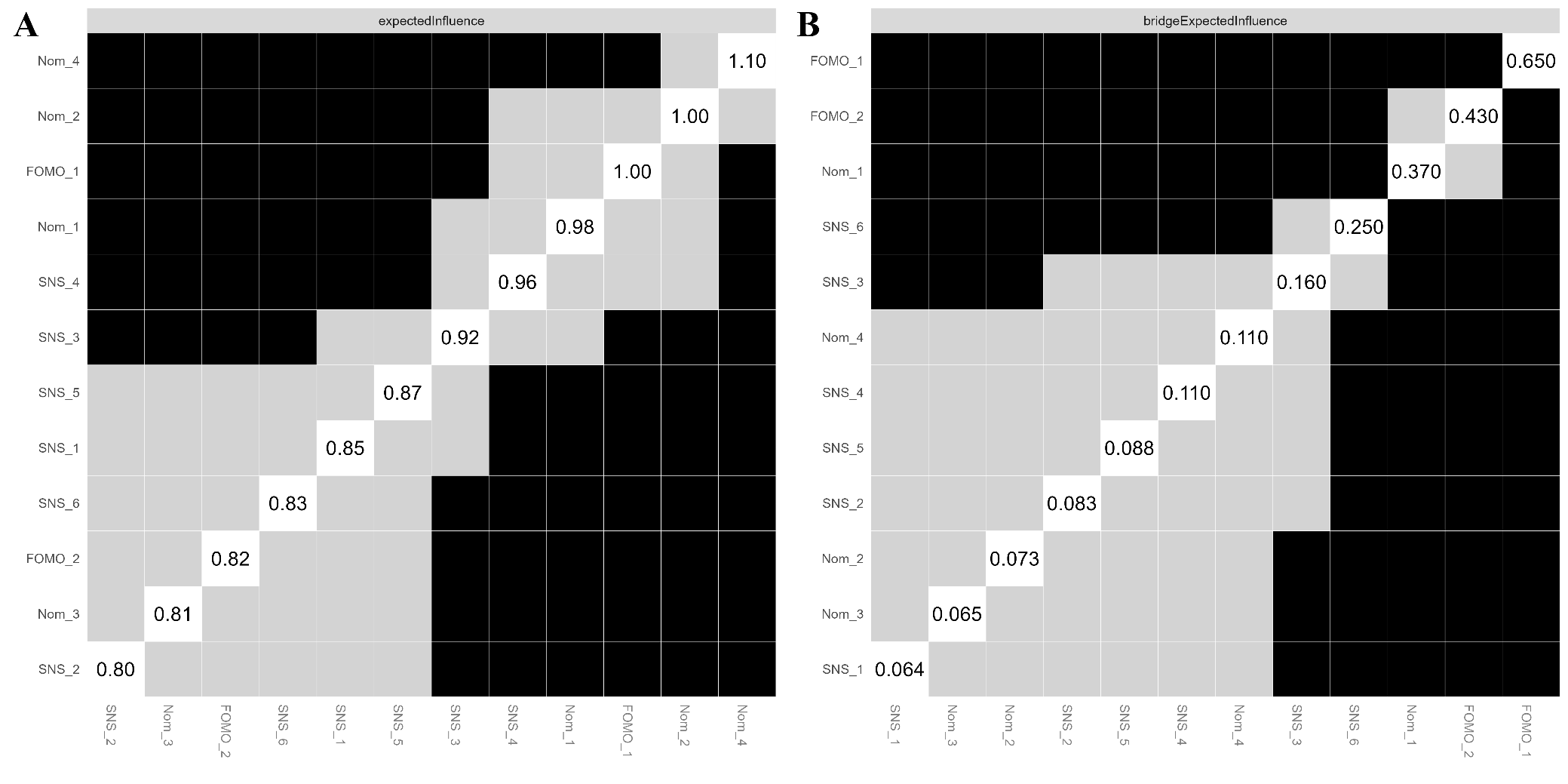


**Figure S4: Bootstrapped difference test for EI and Bridge EI of each node in the network.**

***Note*.** Box colors indicate whether edge weights differ significantly: black denotes significant differences, while gray indicates no significant difference. The numbers in the white boxes (i.e., diagonal line) represent the values of the nodes’ raw centrality.
